# A magnetoencephalography dataset during three-dimensional reaching movements for brain-computer interfaces

**DOI:** 10.1038/s41597-023-02454-y

**Published:** 2023-08-22

**Authors:** Hong Gi Yeom, June Sic Kim, Chun Kee Chung

**Affiliations:** 1 Department of Electronics Engineering, Chosun University, 309 Pilmundae-ro, Dong-gu, Gwangju, 61452 Republic of Korea; 2 Interdisciplinary Program in IT-Bio Convergence System, Chosun University, Gwangju, 61452 Republic of Korea; 3 Clinical Research Institute, Konkuk University Medical Center, 120-1 Neungdong-ro, Gwangjin-gu, Seoul, 05030 Republic of Korea; 4 Interdisciplinary Program in Neuroscience, Seoul National University, Seoul, 08826 Republic of Korea; 5 Department of Neurosurgery, Seoul National University College of Medicine and Hospital, Seoul, 03080 Republic of Korea; 6 Neuroscience Research Institute, Seoul National University Hospital, Seoul, 03080 Republic of Korea

**Keywords:** Brain-machine interface, Neural decoding

## Abstract

Studying the motor-control mechanisms of the brain is critical in academia and also has practical implications because techniques such as brain-computer interfaces (BCIs) can be developed based on brain mechanisms. Magnetoencephalography (MEG) signals have the highest spatial resolution (~3 mm) and temporal resolution (~1 ms) among the non-invasive methods. Therefore, the MEG is an excellent modality for investigating brain mechanisms. However, publicly available MEG data remains scarce due to expensive MEG equipment, requiring a magnetically shielded room, and high maintenance costs for the helium gas supply. In this study, we share the 306-channel MEG and 3-axis accelerometer signals acquired during three-dimensional reaching movements. Additionally, we provide analysis results and MATLAB codes for time-frequency analysis, F-value time-frequency analysis, and topography analysis. These shared MEG datasets offer valuable resources for investigating brain activities or evaluating the accuracy of prediction algorithms. To the best of our knowledge, this data is the only publicly available MEG data measured during reaching movements.

## Background & Summary

Studying the motor-control mechanisms of a brain is critical not only in academia and also has practical implications^1^ because techniques such as brain-computer interfaces (BCIs) can be developed based on brain mechanisms^2^. The BCI is a promising technology that enables people to control electronic devices or express their intentions using brain signals^2–4^. For example, BCI users can move an electric wheelchair based on power changes in the motor cortex^4^. They can also type words or control a robotic arm based on the neural activities of the visual cortex or motor cortex, respectively^4^. Therefore, the BCI is an essential technology for people with motor disabilities and a helpful technology that provides convenient interfaces for healthy people^4^. Thus, multiple studies have investigated brain mechanisms^1,5–9^ and developed the BCIs^4,10^. Despite this, only a small amount of magnetoencephalography (MEG) data has been shared publicly^11,12^. For example, the Human Connectome Project (HCP) dataset (https://db.humanconnectome.org) is a large-scale, open-source dataset consisting of magnetic resonance imaging (MRI) and MEG data from 1,200 young adults aged 22–35 years. However, the HCP dataset contains MEG data from only 67 subjects. The MEG data related to the motor task were two sessions (14 minutes per session) per subject. The motor task was simple finger tapping or toe squeezing.

In contrast, various electroencephalography (EEG) data have been shared to promote EEG-related studies^13–15^. EEG is commonly used for BCI studies because the equipment is relatively cheap and easy to use^13^. However, the EEG’s signal-to-noise ratio (SNR) is quite low because of volume conduction problems caused by the scalp, skull, and cerebrospinal fluid^16,17^. Although electrocorticography (ECoG), local field potential (LFP), or spike show high-quality signals, these methods require surgery. Therefore, measuring healthy subjects’ LFP or spike signals is almost impossible.

In contrast, MEG signals have the highest spatial resolution (~3 mm) and temporal resolution (~1 ms) among the non-invasive methods^3,18,19^, making the MEG an excellent modality to investigate brain mechanisms^19^. A drawback of the MEG is that the equipment is expensive, and the maintenance cost for the helium gas supply is high. Furthermore, a magnetically shielded room is required^18^. For these reasons, obtaining MEG study data is not easy for researchers^11^.

Here, we share the 306-channel MEG and 3-axis accelerometer signals which were measured during three-dimensional (3D) reaching movements. Studying the reaching mechanisms is a critical issue in neuroscience and enables disabled people to control robotic arms like real ones. During the experiment, subjects were instructed to move their arm according to the 3D ball and bar images. An accelerometer on the index finger took simultaneous measurements, along with the MEG signals. The MEG data utilized in this study has previously been employed to investigate the underlying neural mechanisms in motor control^1,5,6^ and to evaluate the performance of algorithms predicting movements^6,20–24^. Therefore, the shared MEG datasets may be used to investigate brain mechanisms using new analytical methods or compare prediction performance for newly proposed algorithms. MATLAB codes to analyse the shared data are also provided for time-frequency analysis, F-value time-frequency analysis^25^, and topography analysis. To the best of our knowledge, this data is the only publicly available MEG data measured during reaching movements.

## Methods

### Participants

Nine subjects (five men and four women) participated in the experiment. Their ages ranged from 19 to 37 years (26.7 ± 6.8). Only right-handed subjects were recruited because they would need to move their right arm during the experiment. Right-handedness was confirmed using the Edinburgh Handedness Inventory scores (above 80)^26^. We confirmed that all participants were not colour-blind because they would observe 3D visual stimuli consisting of blue and red colours. The experimental procedures were approved by the Institutional Review Board of the Seoul National University Hospital (approval number: 1105-095-363). Before commencing the study, all subjects read and signed consent forms about the experiment and were aware that they could stop the experiment at any time. The study was performed in accordance with the Declaration of Helsinki.

### Experimental paradigm

**Fig. 1:**
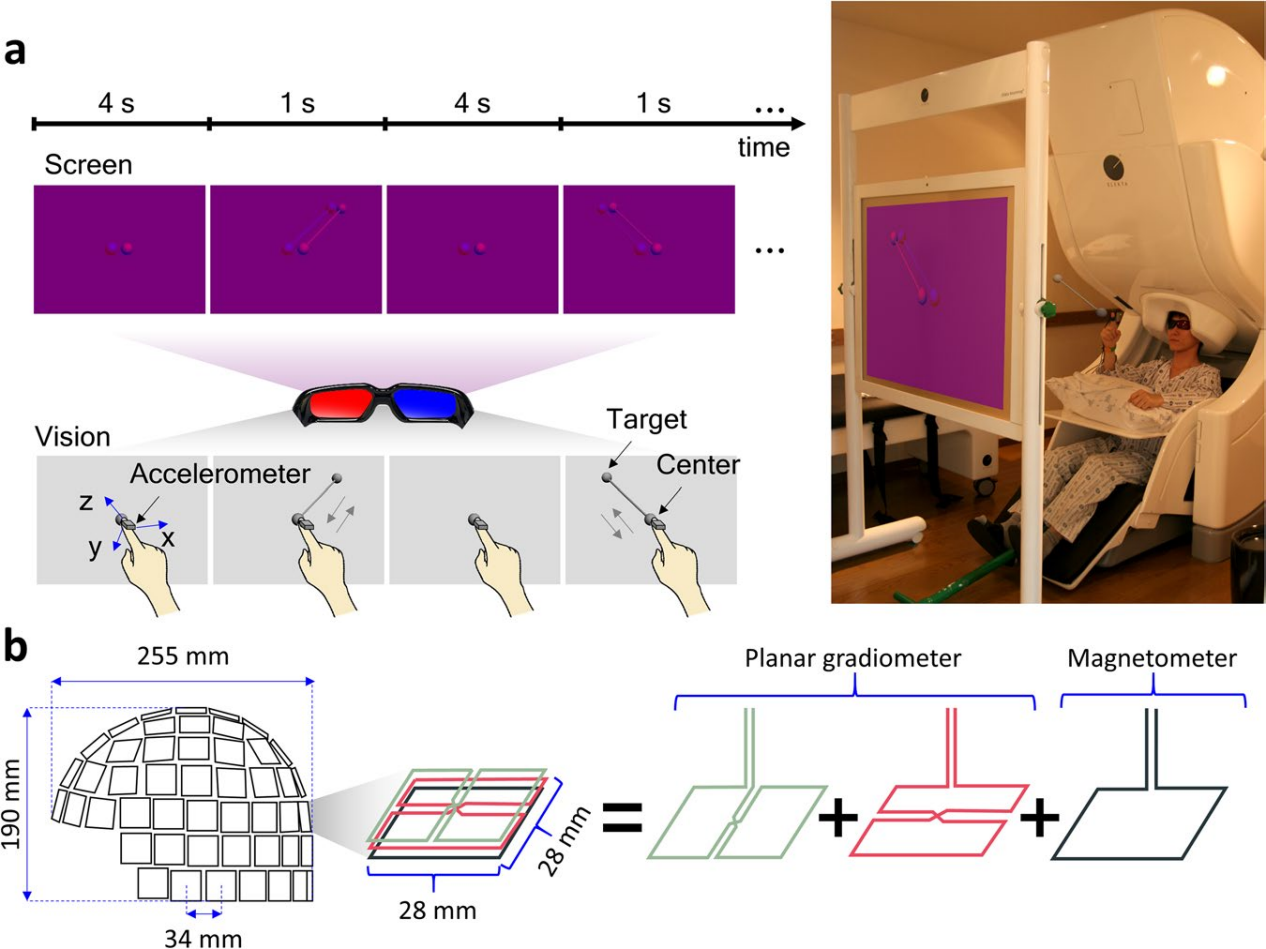
Experimental design. (**a**) Experimental paradigm and acquisition setup. To guide the consistent movements, 3D images were shown on a screen based on an anaglyph method. Subjects were instructed to move their right arm according to the 3D images (centre-out paradigm). The target was sudo-randomly presented among the four corners (upper-left, upper-right, bottom-left, and bottom-right). The subject consented to his image being published. (**b**) Composition of MEG sensors. A whole-head MEG system has 306 sensors at 102 locations. Two planar gradiometers and one magnetometer are located at the same position (102 magnetometers and 204 planar gradiometers).

Figure 1a shows the experimental paradigm and acquisition setup. The participant was seated on a comfortable chair in front of a screen. A cushion was placed under the subject’s elbow to minimize movement artefacts. Visual stimuli were presented on the screen using a projector. To guide consistent movements, 3D images were shown on a screen based on an anaglyph method. Figure 1b depicts the composition of the MEG sensors. A whole-head MEG system has 306 sensors at 102 locations. Two planar gradiometers and one magnetometer are located at the same position (102 magnetometers and 204 planar gradiometers).

The principle of the anaglyph is as follows. A subject wears glasses coloured with a red lens on the left and a blue lens on the right. Images with a red object on the left and a blue object on the right are shown to the subject. At this time, the left eye looking through the red lens, cannot see the red object. Similarly, the right eye looking through the blue lens, cannot see the blue object. Therefore, the left eye sees the right-side object, and the right eye sees the left-side object. As a result, the subject’s brain perceives the object as existing at the intersection of sight.

To make anaglyph images, we created a 3D model using the Autodesk 3ds Max 2011 program (Autodesk, San Rafael, CA, USA) and saved images from two different angles. One angle was the left view, and the other was the right view. Using model images saved from the different view angles, the Anaglyph Maker software (ver. 1.08) then produced anaglyph images. The Anaglyph Maker software is available at http://www.stereoeye.jp.

During the experiment, anaglyph 3D images were presented on the screen to guide consistent movements using a STIM2 system (Neuroscan, El Paso, TX, USA). Subjects were instructed to move their right arm according to the 3D images. They were requested to minimize any other movements. At first, a sphere was presented in the centre of the image for a duration of 4 s. At this time, the subject was required to put an index finger on the centre sphere. Thereafter, a target sphere appeared in a corner with a cylinder connecting the target to the centre for 1 s. At this time, the subject moved their index finger to the target, along the cylinder, and back (a centre-out paradigm). The one-reaching movement was considered as one trial. The process was then repeated and two sessions were measured for each subject. One session consisted of 30 trials for each target (total: 120 trials) and there was a short break between the sessions. The target was sudo-randomly presented among the four directions (upper-left, upper-right, bottom-left, and bottom-right).

### Data acquisition

**Fig. 2:**
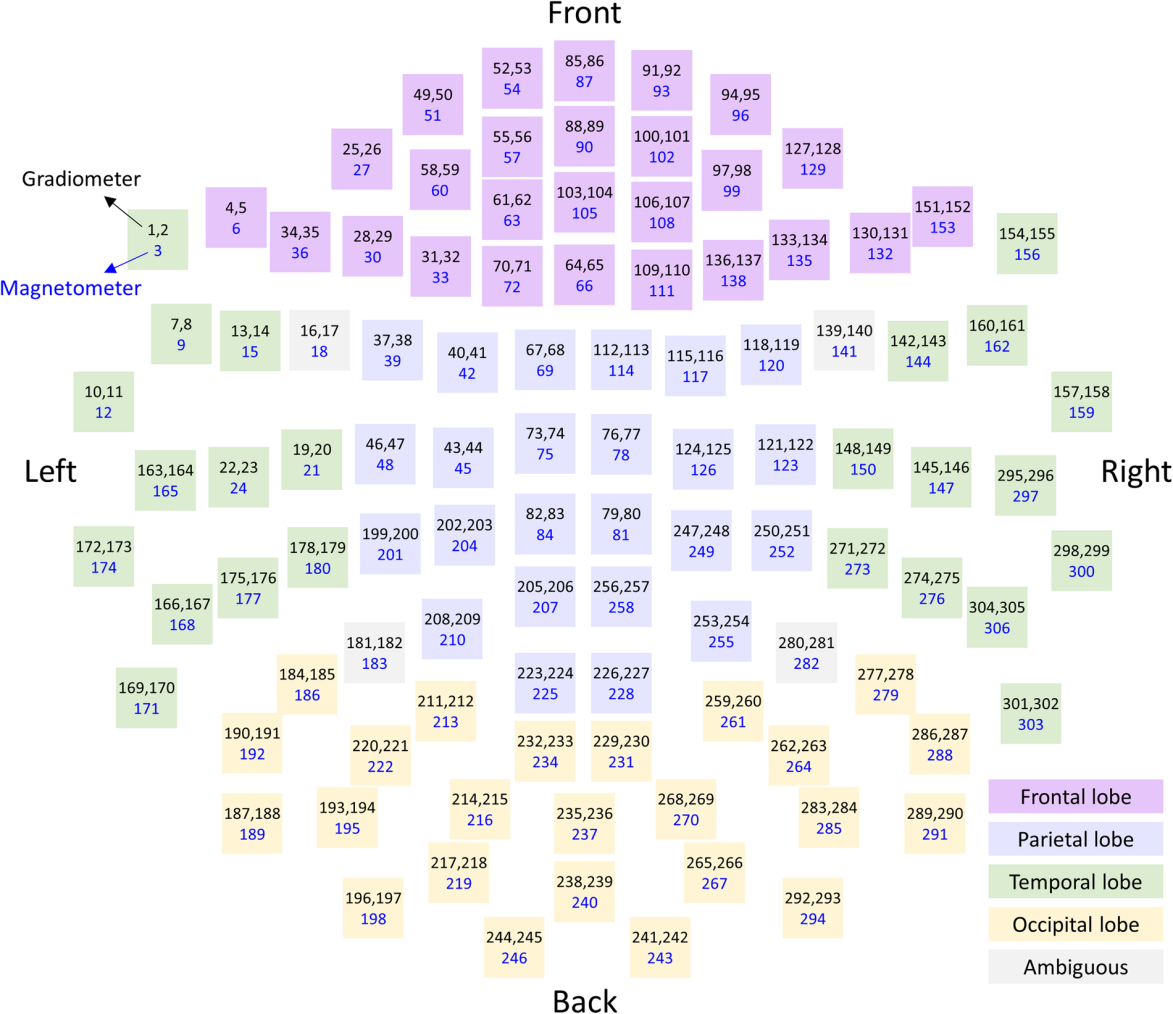
Channel location of MEG sensors. Each square represents the channel location of two planar gradiometers and one magnetometer. Blue numbers refer to magnetometers and black numbers refer to gradiometers. The colour of the square represents the approximate anatomical location.

A 306-channel whole-head MEG system (VectorView TM, Elekta Neuromag Oy, Helsinki, Finland) was used to measure brain activities. The channel locations of the MEG sensors are illustrated in Fig. 2. Each square represents the channel location of two planar gradiometers and one magnetometer. Blue numbers refer to magnetometers and black numbers refer to gradiometers. The colour of the square represents the approximate anatomical location. All experiments were measured in a magnetically shielded room. The sampling frequency was 600.615 Hz, and the signals were band-pass filtered at 0.1–200 Hz. Trajectories of the arm movements were recorded using a three-axis accelerometer (KXM52, Kionix, NY, USA) attached to the index finger. The sensor signals were measured simultaneously with the MEG signals using the same sampling frequency.

### Preprocessing

The spatiotemporal signal space separation (tSSS) method was applied to the MEG signals^27^. The tSSS algorithm removes external interference from MEG signals based on sensor geometry and Maxwell’s equations. Furthermore, the tSSS eliminates artefacts from nearby sources using a statistical method in the time domain. Raw MEG data applied to the tSSS was provided as fif format files. To read the fif file, the MNE MATLAB toolbox is required^28^ and is available at https://github.com/mne-tools/mne-matlab.

Segmented MEG data (epoched data) was also shared as mat format files for convenience. The MEG data were divided into epochs from −1 s to 2 s from the cue onset. The 120 trials for each session were separated into four groups (30 trials in each group) based on the target positions. The four group epochs were saved as cell-type data. All data processing after tSSS was performed using MATLAB R2022a (Mathworks, Natick, MA, USA).

### Artefact removal using ICA

Additionally, we provide MEG data with reduced noise using independent component analysis (ICA). The ICA algorithm calculates the original signals from mixed signals containing multiple components. The “runica” and “topoplot” functions provided by EEGLAB were used to analyse the MEG data and remove components suspected to be caused by eye movements or body movements^29^. The independent components were analysed by plotting topographies from the 102 channels of each type because the MEG equipment consisted of two planar gradiometers and one magnetometer, as shown in Figs. 1b, 2. The artefact components were determined based on the topography pattern. The number of removed components from the two planar gradiometers and one magnetometer were 16 ± 4.34, 12 ± 5.72, and 20.72 ± 5.65, respectively.

## Data Records

### Data format

**Fig. 3:**
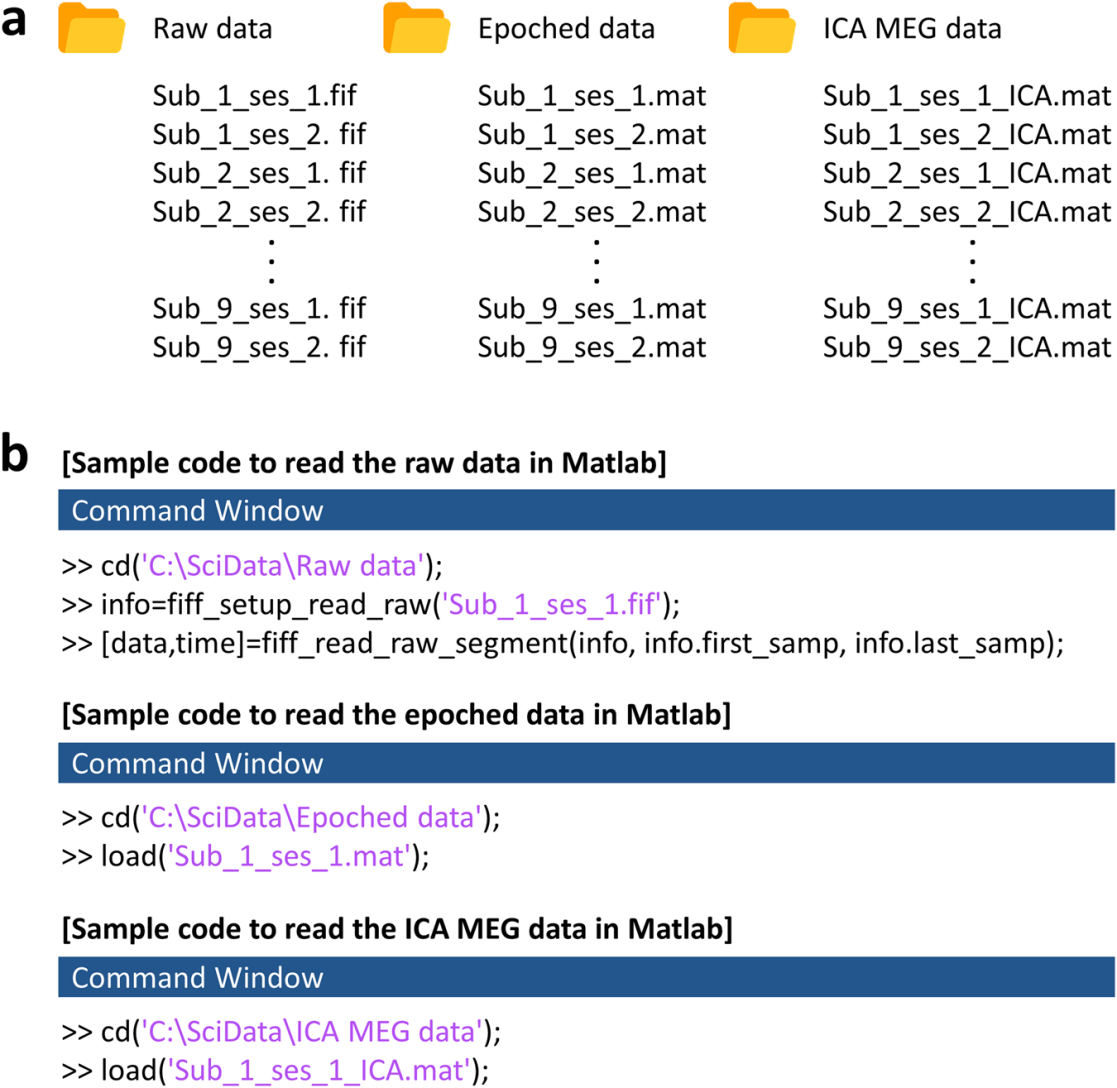
Data structure and sample MATLAB codes. (**a**) The file structures of raw, epoched, and ICA MEG data. The raw data folder has continuous MEG signals after tSSS filtering^26^. The epoched data folder includes MEG signals truncated before and after the events. The ICA MEG data folder has epoched MEG signals after artefact removal using ICA. (**b**) Sample MATLAB codes to read the data. To read the fif file, the MNE MATLAB toolbox is required^27^.

The MEG data is freely available by citing this paper and can be downloaded from figshare^30^. Three types of MEG data were shared. One is the continuous raw MEG data provided in fif format. Another is the epoched MEG data, provided in mat format, and is compatible with MATLAB software. The other is the epoched MEG data after the artefact removal using ICA (ICA MEG data). The file structures of the raw, epoched, and ICA MEG data are illustrated in Fig. 3a. The raw data folder has continuous MEG signals after tSSS filtering and contains 18 files with measurements from nine subjects across two sessions. The epoched data folder includes MEG signals truncated before and after the events. The folder has 18 mat files from nine subjects across two sessions. The folder of the ICA MEG data has the same structure as the epoched data folder. The sample MATLAB codes required to read the data are shown in Fig. 3b.

**Fig. 4:**
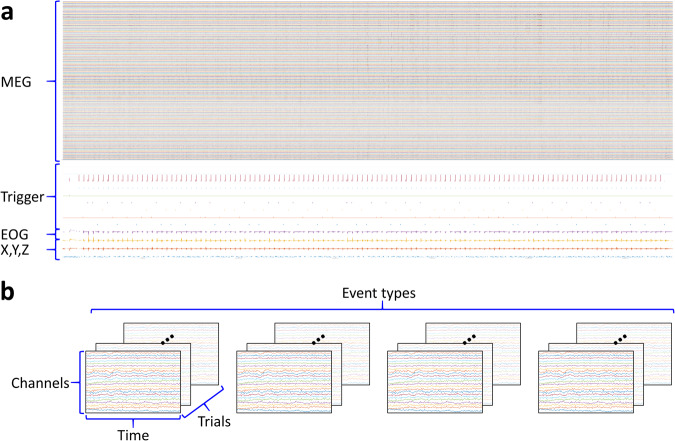
Data shape of the raw and epoched data. Both the raw and epoched data have 319 channels. The 1 ~ 306 channels are MEG signals. The 307 ~ 315 are trigger signals. The 316 is the EOG signal. The 317 ~ 319 are the accelerometer signals on the x, y, and z-axis, respectively. (**a**) Data shape of the raw data. (**b**) Data shape of the epoched data. The data type of the epoched data is a cell array and has four cells. Each cell contains 3D matrix data for one event type (same movement direction). The 3D matrix consists of channels, time, and trials.

These raw, epoched, and ICA MEG data have 319 channels, as shown in Fig. 4a. The 1 ~ 306 channels are MEG signals. The 307 ~ 315 are trigger signals. The 316 is the electrooculography (EOG) signal. The 317 ~ 319 are the accelerometer signals on the x, y, and z-axis, respectively. The data type of the epoched and ICA MEG data is a cell array. The epoched and ICA MEG data have four cells, respectively, as shown in Fig. 4b. Each cell contains 3D matrix data for one event type (same movement direction). The 3D matrix consisted of channels, time, and trials.

## Technical Validation

### Time-frequency analysis

**Fig. 5:**
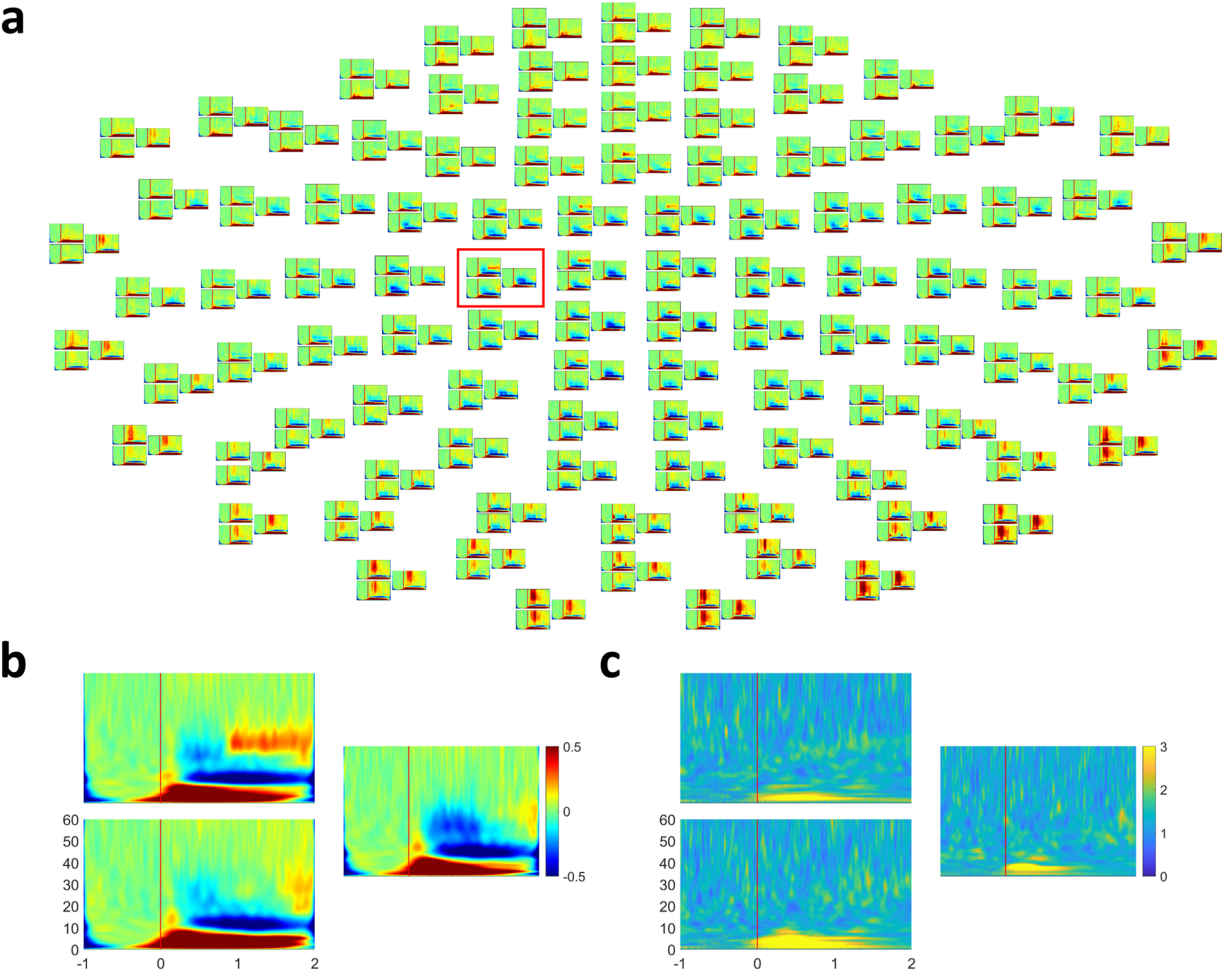
Time-frequency analysis and F-value Time-frequency (FTF) analysis. (**a**) time-frequency analysis on 306 whole channels averaged by all subjects, trials, and event types. The baseline of the spectrum was from −1 to 0 s based on the cue onset. The time-frequency analysis revealed that event-related desynchronization (ERD) is prominent around the motor cortex areas at alpha (8–13 Hz) and beta (13–30 Hz) waves. (**b**) A big size plot of the red box channels in (**a**) Event-related synchronization (ERS) and ERD appeared at 0.1–8 and 9–37 Hz, respectively. ERD changed to ERS at 23–37 Hz after the movements. (**c**) The FTF analysis of the same channels with (**b**) FTF analysis is a statistical analysis method to investigate distinguishing characteristics by applying the F-value of ANOVA to time-frequency analysis^24^. The results represent differences among the different directions of reaching movements and are represented at 1–6 Hz based on the FTF analysis. The results explain why our previous studies could predict arm movements from neural signals filtered at 0.5–8 Hz^6,19,21^.

Figure 5a represents the time-frequency analysis on 306 whole channels averaged by all subjects, trials, and event types. The time-frequency spectra were calculated using a continuous wavelet transform (CWT) at 1 Hz resolution. The power spectrum of each frequency was normalized by each power spectrum of the corresponding baseline^31^. The baseline of the spectrum was from −1 to 0 s, based on the cue onset. Time-frequency analysis revealed that event-related desynchronization (ERD) is prominent around the motor cortex areas at alpha (8–13 Hz) and beta (13–30 Hz) waves. It is well represented on 43–45 channels (red box). Figure 5b is a big size plot of the red box channels. Event-related synchronization (ERS) and ERD appeared at 0.1–8 and 9–37 Hz, respectively. Furthermore, ERD was changed to ERS at 23–37 Hz after the movements and is a well-known characteristic caused by movements^32^.

### F-value Time-frequency analysis

F-value Time-frequency (FTF) analysis revealed the critical neural features for movement prediction, as shown in Fig. 5c. Although time-frequency analysis is a powerful and widely used tool in the analysis of neural activities, it is difficult to identify differences caused by different tasks, such as movement directions, using this method. FTF analysis is a statistical method used to investigate distinguishing characteristics by applying the F-value of ANOVA to time-frequency analysis^25^. FTF analysis visualises the statistical differences among conditions in time-frequency power spectra. The FTF analysis showed a high F-value between 1–6 Hz, as shown in Fig. 5c, and enabled the identification of differences among different directions of reaching movements. The results explain why our previous studies could predict arm movements from neural signals filtered at 0.5–8 Hz^6,20,22^. The MATLAB code of the FTF analysis is freely available at https://github.com/honggi82/FTF-analysis.

### Topography

**Fig. 6:**
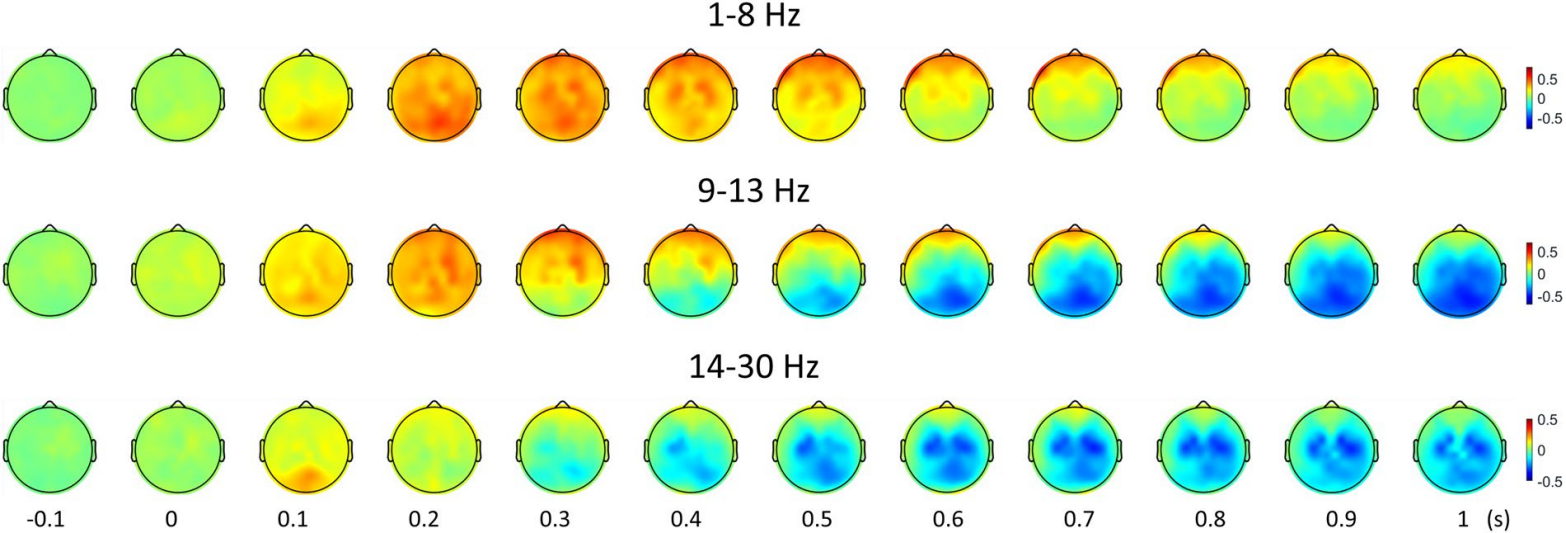
Topography for each frequency band. The topographies illustrate the power change according to the time from −0.1 to 1 s based on the cue onset and reveal the topographical ERD and ERS patterns. ERD occurred at a more specific area at the beta band (14–30 Hz) than at the alpha band (8–13 Hz). The topography at the beta band revealed that ERD was generated on the contralateral arm area and it was propagated to the ipsilateral area.

Figure 6 illustrates topography according to the time for each frequency band from −0.1 to 1 s based on the cue onset and shows the topographical ERD and ERS patterns. ERD occurred in a more specific area at the beta band (14–30 Hz) than at the alpha band (8–13 Hz). The topography at the beta band revealed that ERD was generated on the contralateral arm area, and it was propagated to the ipsilateral area. These results correspond to the previous studies^32^. The topography was calculated by averaging the power of the gradiometer’s corresponding frequency band, from the time-frequency spectra, for each time point.

### Movement prediction

To demonstrate the predictability of movements from shared MEG data, we utilized bidirectional long-short term memory (Bi-LSTM) to predict arm movements from the MEG signals. Initially, we applied Independent Component Analysis (ICA) to eliminate eye and movement artefacts from the preprocessed MEG data. From the 306 MEG channels, we selected 68 gradiometer channels located in brain regions associated with motor control. Subsequently, these 68 channels were band-pass filtered at 0.5–8 Hz and downsampled to 50 Hz. As the arm’s position was measured using an accelerometer, velocity was calculated by integrating accelerometer signals. To mitigate noise in the velocity data, we performed band-pass filtering at 0.5–10 Hz before and after the integral process. The filtered velocity signals were then downsampled to 50 Hz. We divided the downsampled MEG and velocity signals into training and test datasets using a 5-fold cross-validation approach to prepare the data for model training. Before training the model, we normalized the MEG and velocity signals.

**Fig. 7:**
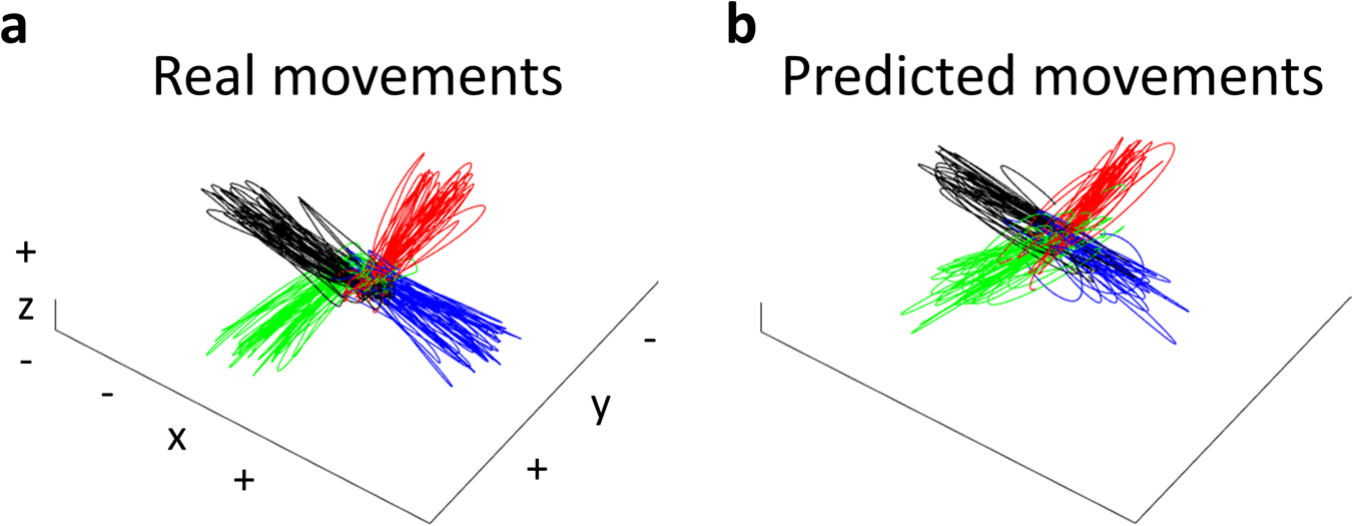
Movement prediction results from the MEG signals for one session of one subject. (**a**) Real movement trajectory. (**b**) Predicted movement trajectory from the MEG signals. Each colour indicates the movement direction. Distance unit is arbitrary because it is calculated from accelerometer signals.

The architecture of the prediction model included a sequence input layer, a Bi-LSTM layer, a fully connected layer, a dropout layer, another fully connected layer, and a regression layer. The Bi-LSTM layer had 200 hidden units. The Bi-LSTM model was trained with the training data. For the training, we set the maximum number of epochs to 60 and the mini-batch size to 20. The Bi-LSTM model’s predictive performance was evaluated using the test data. The accuracy of the predictions was assessed by calculating Pearson’s correlation coefficients (*r*) between the real and predicted movements. The mean correlation coefficients averaged across all subjects were 0.714 ± 0.088, 0.749 ± 0.073, and 0.798 ± 0.066 (mean ± SD) for the x-axis, y-axis, and z-axis, respectively (*p* < 0.001). Figure 7 shows the real and predicted movement trajectories for one session of one subject. The results indicate that the predicted movements from MEG signals are very similar to the real movements. We applied band-pass filtering at 0.5–10 Hz, removal of a linear trend, and integral to calculate the position from velocities in Fig. 7. These signal-processing methods were determined based on our previous studies^6,20,22^.

### Limitations and alternatives

Although our previous studies utilizing the shared data revealed the neural mechanisms^1,5,6^ and successfully trained the deep learning algorithms^6,22^, the dataset may not be sufficient for training super-giant AI models like the generative pre-trained transformer (GPT). The issue of data scarcity can be partially overcome by data augmentation^33,34^, generation^35^, and transfer learning^36^ techniques. Data augmentation involves slight modifications to the existing data to increase its quantity, achieved through methods such as noise addition, sliding windows, and recombination of segmentation. Careful attention is required during data transformation to preserve the original data’s characteristics. The data generation method utilizes generative adversarial network (GAN) algorithms to learn data distributions and generate synthetic data that resembles the original data. However, it is required to acknowledge that GAN-generated synthetic data may not fully replicate the properties of the original data. Lastly, transfer learning involves retraining pre-existing models using user data. Transfer learning enables achieving good performance even with limited data. The pre-trained model performs better when its training data shares similar characteristics to user data.

## Usage Notes

The MNE toolbox is a prerequisite to reading the raw MEG data, as described above. The MNE MATLAB files can be downloaded at https://github.com/mne-tools/mne-matlab. A “topoplot” function and related sub-functions in the EEGLAB toolbox are needed to plot a topography. The EEGLAB software is available at https://sccn.ucsd.edu/eeglab 29. For time-frequency analysis or FTF analysis, our custom MATLAB codes are required. These can be found at https://github.com/honggi82/Scientific_Data_2023. To use these toolboxes, the folders containing the toolboxes should be added to the “Set Path” of MATLAB.

The “Out of memory” error could occur when MEG data is processed because the data size is large. To this end, we recommend using a computer with a large amount of RAM and using 64-bit MATLAB and a 64-bit operating system (OS). Increasing the virtual memory and clearing unnecessary variables during the analysis is also recommended. We used Intel(R) Core(TM) i9-7900 × 3.30 GHz CPU, 64 GB RAM, a 64-bit Windows 10 OS, and a 64-bit MATLAB R2022a for the study.

## Data Availability

The MATLAB scripts are available for loading and analysing data under the MIT License at https://github.com/honggi82/Scientific_Data_2023.

## References

[1] Yeom HG, Kim JS, Chung CK (2020). Brain mechanisms in motor control during reaching movements: Transition of functional connectivity according to movement states. Sci Rep.

[2] Dornhege, G. *Toward brain-computer interfacing*. (MIT Press, 2007).

[3] Nam, C. S., Nijholt, A. & Lotte, F. *Brain-computer interfaces handbook: technological and theoretical advances*. (Taylor & Francis, CRC Press, 2018).

[4] Choi WS, Yeom HG (2022). Studies to Overcome Brain-Computer Interface Challenges. Appl Sci-Basel.

[5] Yeom HG, Kim JS, Chung CK (2016). Macroscopic Neural Oscillation during Skilled Reaching Movements in Humans. Comput Intel Neurosc.

[6] Kim H, Kim JS, Chung CK (2023). Identification of cerebral cortices processing acceleration, velocity, and position during directional reaching movement with deep neural network and explainable AI. Neuroimage.

[7] Yang YJ, Jeon EJ, Kim JS, Chung CK (2021). Characterization of kinesthetic motor imagery compared with visual motor imageries. Sci Rep.

[8] Schwartz AB (2016). Movement: How the Brain Communicates with the World. Cell.

[9] Sun XL (2022). Cortical preparatory activity indexes learned motor memories. Nature.

[10] Willett FR, Avansino DT, Hochberg LR, Henderson JM, Shenoy KV (2021). High-performance brain-to-text communication via handwriting. Nature.

[11] Rathee D, Raza H, Roy S, Prasad G (2021). A magnetoencephalography dataset for motor and cognitive imagery-based brain-computer interface. Sci Data.

[12] Hodge MR (2016). ConnectomeDB–Sharing human brain connectivity data. Neuroimage.

[13] Lee YE, Shin GH, Lee MJ, Lee SW (2021). Mobile BCI dataset of scalp- and ear-EEGs with ERP and SSVEP paradigms while standing, walking, and running. Sci Data.

[14] Nieto N, Peterson V, Rufiner HL, Kamienkowski JE, Spies R (2022). Thinking out loud, an open-access EEG-based BCI dataset for inner speech recognition. Sci Data.

[15] Won K, Kwon M, Ahn M, Jun SC (2022). EEG Dataset for RSVP and P300 Speller Brain-Computer Interfaces. Sci Data.

[16] Boenstrup M, Feldheim J, Heise K, Gerloff C, Hummel FC (2014). The control of complex finger movements by directional information flow between mesial frontocentral areas and the primary motor cortex. Eur J Neurosci.

[17] Singh SP (2014). Magnetoencephalography: Basic principles. Ann Indian Acad Neur.

[18] Holmes N (2022). A lightweight magnetically shielded room with active shielding. Sci Rep.

[19] Baillet S (2017). Magnetoencephalography for brain electrophysiology and imaging. Nat Neurosci.

[20] Yeom HG, Kim JS, Chung CK (2013). Estimation of the velocity and trajectory of three-dimensional reaching movements from non-invasive magnetoencephalography signals. J Neural Eng.

[21] Yeom HG, Kim JS, Chung CK (2014). High-Accuracy Brain-Machine Interfaces Using Feedback Information. Plos One.

[22] Yeom HG, Kim JS, Chung CK (2020). LSTM Improves Accuracy of Reaching Trajectory Prediction From Magnetoencephalography Signals. Ieee Access.

[23] Yeom HG (2014). A Study on Decoding Models for the Reconstruction of Hand Trajectories from the Human Magnetoencephalography. Biomed Res Int.

[24] Kim YJ (2015). A study on a robot arm driven by three-dimensional trajectories predicted from non-invasive neural signals. Biomed Eng Online.

[25] Yeom HG, Jeong H (2021). F-Value Time-Frequency Analysis: Between-Within Variance Analysis. Front Neurosci.

[26] Oldfield RC (1971). The assessment and analysis of handedness: the Edinburgh inventory. Neuropsychologia.

[27] Taulu S, Simola J (2006). Spatiotemporal signal space separation method for rejecting nearby interference in MEG measurements. Phys Med Biol.

[28] Gramfort A (2014). MNE software for processing MEG and EEG data. Neuroimage.

[29] Delorme A, Makeig S (2004). EEGLAB: an open source toolbox for analysis of single-trial EEG dynamics including independent component analysis. J Neurosci Methods.

[30] Yeom HG, Kim JS, Chung CK (2023). A magnetoencephalography dataset during three-dimensional reaching movements for brain-computer interfaces.

[31] Rickert J (2005). Encoding of movement direction in different frequency ranges of motor cortical local field potentials. J Neurosci.

[32] Pfurtscheller G, Lopes da Silva FH (1999). Event-related EEG/MEG synchronization and desynchronization: basic principles. Clin Neurophysiol.

[33] Fan J (2020). EEG data augmentation: towards class imbalance problem in sleep staging tasks. J Neural Eng.

[34] Lashgari E, Liang D, Maoz U (2020). Data augmentation for deep-learning-based electroencephalography. J Neurosci Methods.

[35] Habashi AG, Azab AM, Eldawlatly S, Aly GM (2023). Generative adversarial networks in EEG analysis: an overview. J Neuroeng Rehabil.

[36] Zhang, K. *et al*. Application of Transfer Learning in EEG Decoding Based on Brain-Computer Interfaces: A Review. *Sensors (Basel)* **20**, 10.3390/s20216321 (2020). 10.3390/s20216321 PMC7664219 33167561

